# Policies and Toxicological Screenings for No Drug Addiction: An Example from the Civil Aviation Workforce

**DOI:** 10.3390/ijerph19031501

**Published:** 2022-01-28

**Authors:** Michele Treglia, Margherita Pallocci, Giorgio Ricciardi-Tenore, Flavio Baretti, Giovanna Bianco, Paola Castellani, Fabrizio Pizzuti, Valeria Ottaviano, Pierluigi Passalacqua, Claudio Leonardi, Luca Coppeta, Agostino Messineo, Roberta Tittarelli

**Affiliations:** 1 Section of Legal Medicine, Social Security and Forensic Toxicology, Department of Biomedicine and Prevention, University of Rome “Tor Vergata”, 00133 Rome, Italy; michelemario@hotmail.it (M.T.); valeryotta@hotmail.com (V.O.); p.passalacqua92@gmail.com (P.P.); afrancisco46@hotmail.it (A.M.); roberta.tittarelli@gmail.com (R.T.); 2 Department of Biomedicine and Prevention, University of Rome “Tor Vergata”, 00133 Rome, Italy; giorgio.ricciarditenore@gmail.com (G.R.-T.); baretti.flav@tiscali.it (F.B.); giovannabianco70@gmail.com (G.B.); castellanipaola58@gmail.com (P.C.); fabriziopizzuti@hotmail.com (F.P.); lcoppeta@gmail.com (L.C.); 3 Substance Abuse and Mental Health Services (Ser.D)—Local Health Authority (ASL) RM2, 00133 Rome, Italy; leonardiclaudio1958@libero.it

**Keywords:** substance-related disorders, substance abuse, toxicology, occupational health, occupational medicine, health policy

## Abstract

Introduction: Since 2008, Italian legislators, with the aim of ensuring public safety, have made it mandatory for an occupational doctor (OD) to assess specific categories of workers to exclude those who may have consumed drugs of abuse. Due to the relevance of work activities relating to the civil aviation and airport sector, a policy based on the use of training and information tools, as well as a health surveillance protocol, has been undertaken since 2009. Materials and methods: A total of 61,008 workers at a commercial airline underwent health surveillance between 2009 and 2019. Following ≤24 h notification, their urine was screened for opiates, cocaine, cannabinoids, amphetamines, methamphetamines, and methylenedioxymethamphetamine (MDMA) using an immunochemical test. Positive results were confirmed using Gas Chromatography-Mass Spectrometry (GC/MS) or Liquid Chromatography -Mass Spectrometry (LC/MS). In confirmed cases, the workers were declared unfit and sent to a specialized laboratory for a second level analysis. Results: Positive results, initially >1%, have halved in the last four years (<0.5%). The percentage of positive results was consistently very low among pilots and, moreover, the rare positive cases detected were due to a cross-reactivity phenomena. The highest and most discontinuous percentages seen occurred in the population undergoing a pre-employment examination. Regarding the types of substance used, a prevalence of cannabis (58.52%) and cocaine (35.2%) use was observed. Conclusions: The data presented indicate that the air transport sector, in all its components (ground workers and air crews), has a very limited number of substance abusers, and this number tends to decrease over time and with work seniority. Another aspect of particular interest, and which is more specific to toxicology, concerns the detection of cross-reactivity in urinary immunochemical screening between the antibodies to drugs of abuse and certain other drugs, such as anti-inflammatories or antibiotics; as well as foods, and other commonly used substances.

## 1. Introduction

Alcohol and substance abuse is a well-known major risk factor for disability and death worldwide. Apart from the social and human costs, addictive behaviors are accompanied by significant economic costs, loss of productivity, and other direct and indirect costs [1].

For a long time, occupational health surveillance has paid close attention to all those duties involving third-party unsafe and unhealthy work-related factors. The practice of Workplace Drug Testing (WDT) began in the United States—following the Drug-Free Workplace Act of April 1988—and is currently performed in government, all areas of transport, and in many of the top companies [2]. In addition, the practice was started in the same year in Europe [3].

Italian legislators have issued various provisions, such as Art. 125 of the Presidential Decree 309/90; Legislative Decree 81/2008, (specifically Art. 41, paragraph 4); State-Regions Conference Provision, dated 30th October 2007; and the State-Regions Conference Agreement of 18 September 2008; following which, some regional regulations have been enacted (i.e., Lazio Regional Council Decision no. 332/09) [4,5,6,7,8,9].

According to the legislation in force in Italy, WDT includes two levels of monitoring: a first stage concerning the drug testing of urine samples, and a second involving both urine and hair analysis. The second stage is performed only on workers who test positive at the first level, to distinguish between sporadic, occasional, or continuative drug abuse.

It should be noted that the current Italian legislation does not provide a specific indication on which analytical method should be used. However, it does require that the results are automatically produced and printed, and that the sensitivity of the test used is higher than the cut-offs reported by the legislator [10].

In the past few years, this complex and specific set of rules has represented something new in terms of health surveillance, with both the introduction of company policies and specific workforce prohibitions, as well as the possibility of an occupational doctor (OD) being employed to detect potentially dangerous behaviors in specific categories of workers, such as their own and third-party safety if under the effect of drugs, psychoactive substances and/or alcohol [5].

From 2009, due to the nature of the aviation industries activities, the airline participating in this study adopted a policy that included the use of specific training and information tools, and a health protocol that investigated the absence of drug or psychoactive substance addiction as well as prohibited alcohol consumption among flight attendants and pilots.

One of the aims of the present study was to investigate the Italian situation in comparison with other countries; also considering that published surveys on air crews and other European workers groups are currently quite limited [11,12,13,14,15].

This report shows the results of the toxicological screen tests carried out from 2010 to 2019 relating to the absence of drug addiction: of the 61,008 preliminary immunochemical screening tests, 614 resulted in positives that required a confirmatory drug test (1%) and only 424 (0.7%) were true positives.

## 2. Materials and Methods

In order to investigate compliance with the policies adopted, as well as any critical problems within the airline workforce framework, the resultant data from medical checks carried out at a civil and commercial airline was examined, from 2010 until 2019. During the preliminary phase, all of the duties involved were analyzed and classified to identify those particularly hazardous to themselves and third-party safety (Table 1).

The training, information, and consultation method involving operators, doctors, and Workers’ Safety Representatives was also evaluated, regarding the application of the health protocol and anti-drug policies-related procedures. The health protocol, provided by the Risk Assessment Document (RAD) included a training phase to make the workers aware of their role. This task was carried out using informative documentation and a publication on safety and against alcohol and drug misuse. The publication was delivered and explained to workers at the same time as the medical examination, and workers were also invited to consult the notices published on the company intranet as well as the posters displayed in the most attended areas. The medical checks relating to health surveillance involved ground and flight attendants and workers in a pre-work assignment (selection), as shown in Table 1.

In 2016, following a reassessment of RAD, the cabin crew (flight attendants), who were previously not subjected to this health surveillance protocol, were also included in the medical checks, as they are in-flight first aid officers with emergency tasks strictly connected to third-party safety.

The assessments on the toxicological screen tests for the absence of drug addiction were carried out in compliance with the regulations of the region where the company is located [9].

The assessment procedures for the periodic, usually annual, medical examination were based on the unpredictability of the date of the medical tests, the short notice of the doctor’s appointment, and the random selection of the worker. At each medical examination, the following was carried out: a specific medical history was taken (e.g., verification of previous driving license withdrawal; treatments and/or hospitalizations for pathologies related to drug or alcohol abuse; a physical examination was performed, aimed at showing any physical and/or psychological signs of misuse); documents were filled in and delivered (information sheets and consent forms, a brochure on safety against drug and alcohol misuse); and a urine test kit was issued at each check (respecting gender and privacy), documented via a countersigned sampling report and the execution of the rapid urine test of level 1 screening.

The collection of urine (at least 60 mL) was always carried out in the presence of the physician or an authorized health professional using a maximum-safety tamper-evident kit that included three urine collection cups, of which the first (A) was used for the collection of the urine sample and the execution of the rapid immunochemical test, on site.

In accordance with the standard protocol [7,8,9,16], and in the presence of the involved party, the specimen was shared among the two other cups of the kit: cup (B) for the possible confirmatory drug test, and cup (C) for the review testing if requested by the worker; these were sealed and countersigned by the healthcare professional and by the person concerned.

For the rapid immunochemical test, a container (Alere™ Drug Screen Urine Test Cup) is used with a multi-parameter test panel for the qualitative analysis of drug abuse and anti-adulteration tests. The test includes a procedure check.

The determinations on the test panel with the relative cut-off values are shown in Table 2.

The test container used is equipped with a thermometric strip for checking the temperature of the urine collected and tests (oxidants/pyridinium chlorochromate, specific gravity, pH, nitrite, glutaraldehyde, and creatinine) for the detection of urinary adulteration.

The results interpretation has been conducted with an instrumented test system with DxLINK Technology: an image of the screening device is electronically captured by the scanner. The test system software analyzes the test controlling line reactivity and yields qualitative test results. The results displayed on the screen will then be saved, sent, and printed.

Preliminary urine screening tests are considered positive if they exceed the cut-off concentration expressed in ng/ml, as reported in Table 2.

The rapid urine drug test enabled the OD to quickly manage the result obtained and issue a work fitness statement in the same session.

- For negative screenings, the OD immediately handed the fitness certificate to the worker, along with a copy of the sampling report and the screening test report;
- For positive screenings, the OD temporarily suspended (10 days) the worker from all third-party risk duties and informed the employer, in writing, of his or her temporary unfitness to work. The “alleged positive” samples, divided into B (for confirmatory drug test) and C (for revision analysis), were sealed and signed by both the OD and the worker, before being sent to a qualified and/or experienced and accredited forensic toxicology laboratory to undergo a confirmatory drug test, using the more selective and sensitive quantitative method of mass spectrometry combined with a chromatographic separation technique (gas or liquid chromatography): GC/MS or LC/MS.

The results of the preliminary screening tests were confirmed based on the cut-off concentration for each substance as shown in Table 2.

In the case of a negative confirmatory test, the OD issued a fitness to work statement resulting in an immediate reinstatement of the worker to his or her duties.

**Figure 1:**
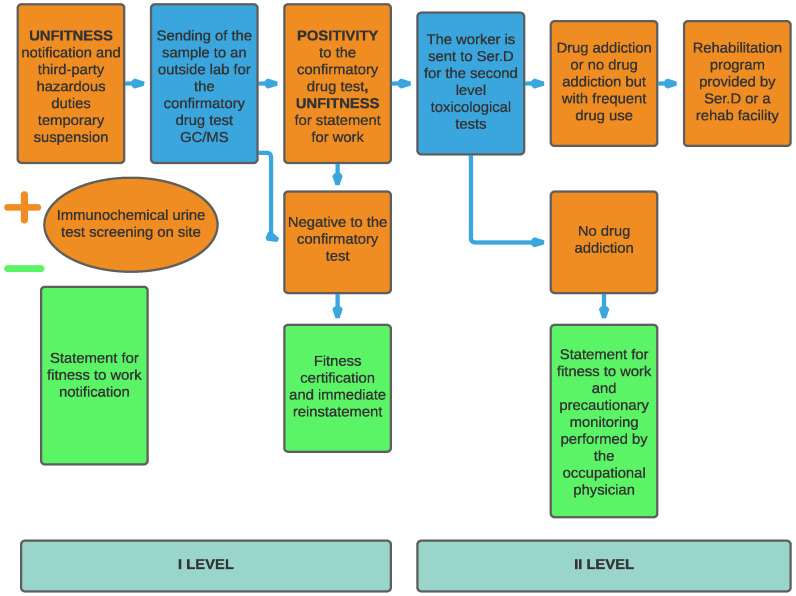
Procedure for issuing the fitness or unfitness statement by the occupational doctor (OD).

In the case of a positive result, the OD issued a new statement of unfitness, notifying both the employer and the worker of such. In this case, a second phase of assessment started, whereby, the worker was sent to a health facility such as Substance Abuse and Mental Health Services (in Italian, Ser.D) or another competent and authorized health facility to carry out level 2 clinical and toxicological or analytical checks, which form the basis of diagnosis using the presence or absence of drug addiction. Once the Ser.D carried out their own evaluation and certified the diagnosis, this was submitted to the OD. This path, shown in Figure 1, has been previously used in other surveys conducted in Italy [17,18], and is valid for all workers apart from pilots, for whom any notification of their positivity was sent to the ENAC (the Italian National Agency for Civil Aviation) [19].

## 3. Results

**Figure 2:**
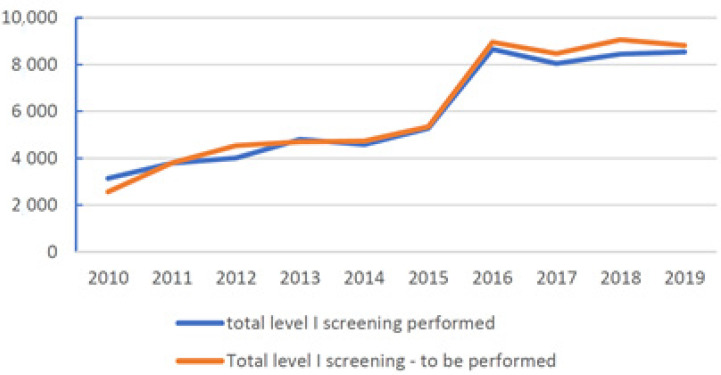
Trend of level 1 screenings carried out from 2010 to 2019.

Figure 2 shows the trend of level 1 screenings carried out from 2010 to 2019 compared to the total number of workers who underwent a toxicological screen test.

The substantial increase observed since 2016 is due to the follow-up that also involved the cabin crew (flight attendants), which amounted to approximately 3500 units/year.

The difference in relationship between the tests carried out and those yet to be carried out is due to job changes, employment termination due to resignations, dismissals, retirements, deaths, or precautionary screenings decided by the OD.

**Figure 3:**
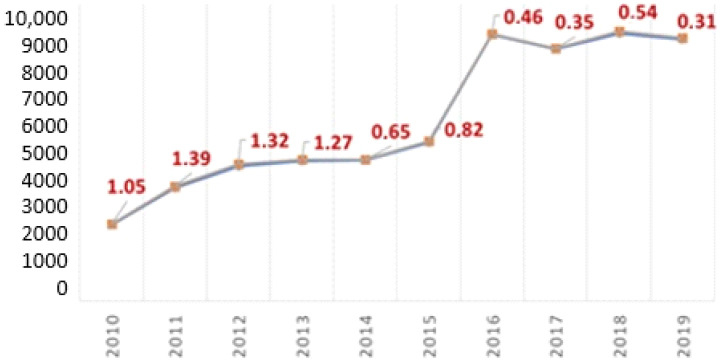
Percentage of positive tests over 10 years (general data).

Conversely, the following diagram shows the number of positive tests and their percentages detected over time as general data (Figure 3). Table 3 shows the groups of worker populations investigated.

The following Table 4 shows the number of positive specimens divided by type of substance, with the percentage of confirmed rate.

It should be noted here that the seeming numerical discrepancy with the above totals in Table 4 and Table 5 is due to samples with multiple positivity.

The percentage, calculated over the entire period considered (2010–2019), showed a prevalence of cannabis (58.52%) and cocaine (35.2%) consumption in analogy with what is also reported in the annual reports to the Italian Parliament, by the National Observatory, the European Drug Report 2017, and the European Monitoring Centre for Drugs and Drug Addiction (EMCDDA) [20,21].

## 4. Discussion

This epidemiological study has highlighted the crucial importance of the use (specifically required by the legislator) of a confirmatory test for the “presumed positive” results obtained via immunochemical screenings. Indeed, the first level assessment of fitness or unfitness of a worker requires the rapid test to be confirmed by a more specific and sensitive method, such as mass spectrometry (MS) combined with gas or liquid chromatography (GC-MS or LC-MS).

Table 4 highlights the total number of samples testing “presumed positive” on the rapid test and those confirmed by GC-MS or LC/MS over the 10 years of analysis, along with the detected percentage. The average rate of confirmed samples was 69%, thus determining the rate of screening test false positives to be about 31%. This finding is in line with the results of other analyses that have reported a prevalence of false positives of 30% [22].

In fact, rapid screening tests for some drug classes (especially amphetamine and methamphetamine) are more likely to test false positive by cross-reacting with molecules other than those for which they were created; unlike others (cocaine, cannabinoids, methadone), which cross-react less frequently.

It should be noted that, among the substances capable of causing a cross-reaction, and listed in recent studies [23,24], in our experience, particular interest was raised by some categories of common antibiotic drugs. The ingestion of medications containing opium poppy husks or the intake of seasonings (e.g., certain packaged seasoned French fries) may also result in a “false positive” urine screening test result [25].

A follow-up on the cross-reactivity of level 1 rapid immunochemical tests will be the subject of a further specific investigation.

As regards to the ground workers, a decreasing trend in positivity percentage was noted; likely as a result of the trainings carried out, the ensuing greater consciousness of his or her own role following the training received, and greater awareness due to the regularly performed medical checks. The initial positivity percentage of > 1% has halved in the last four years (<0.5%). The highest and most discontinuous percentages, in fact, were found in the population subjected to a pre-employment screening (staff recruitment). These are younger people (20–30 years old), mostly at their first work experience, and with poor awareness of their future role within a production cycle. The higher rate of positives resulting from pre-employment visits is a significant finding already highlighted by other authors [26].

With regard to the cabin crew (CC), who only came under the medical checks protocol from 2016,the detected positive results were due to the actual intake of narcotic substances, such as Delta 9-Tetrahydrocannabinol (THC) and cocaine.

On the other hand, the positivity percentage was consistently lower for pilots, and the very limited number of cases testing positive over the 10 years of observation was attributable to the intake of food (poppy seeds) or prescribed drugs or substances whose cross-reactivity has been well demonstrated.

With reference to what has been pointed out by other authors, a study estimated the overall prevalence of positivity resulting from WDT screening tests among Italian workers and evaluated the percentage of true and false positives using confirmatory analysis. In this context, the systematic review and meta-analysis of the scientific literature on WDT in Italy during the period from January 2008 to March 2015 showed an overall prevalence of positivity among Italian workers of 1.4% [95% confidence interval (CI) = 1.1–1.7%], with a decreasing trend over time (probably related to the effects of the pertinent legislation coming into force). Positivity was significantly lower among workers screened with an on-site test (1%; 95% CI = 0.5–1.5%), compared with a bench top test (1.7%; 95% CI = 1.3–2.1%) [22]. In Italy, therefore, the number of true positives to first level drug tests in the workplace appears to be substantially low.

It should be noted that the homogeneity of the cultural and regulatory background between the studies analyzed in the abovementioned review and the one described in the present work is of particular value.

On the other hand, a clear disparity emerges in terms of the percentage of positivity compared with that reported in studies conducted abroad, with extremely variable positivity rates, between 2 and 30% [27,28].

In France, a survey conducted on 1000 truck drivers in the period 2003–2004 using urine tests (with confirmation of the positives via gas chromatography) showed a positive result for cannabinoids in 8.5% of the samples tested, for opioids in 4.1%, for amphetamines in 0.3%, for buprenorphine in 1.8%, for methadone in 0.5%, and for the benzodiazepines in 0.4% [26]. The percentage of positivity found is, therefore, significantly higher than that found in our study. This difference is not explicable on the sole basis of cultural and/or regulatory differences. However, it should be noted that in the work just mentioned, the authors did not specify the adopted sampling protocol (for example, the notification time is not reported), and sampling was carried out anonymously. In addition, other factors may result in a higher rate of positivity. For example, the number and quality of investigated psychotropic substances that differ between countries. It has been also suggested that age, gender, time of day when the sample is taken (higher rates from 6–9 am and 2–5 pm) and type of work are factors associated with a higher positivity rate [29].

In another survey conducted in Italy, in 2012, the first level testing of approximately 92,000 workers (of which 42,866 were in the railway sector) showed a drug test positivity between 0.23% and 0.24% of the subjects tested (with a greater prevalence in the 24–34-year age group), while at the pre-employment examination the positivity was 0.93% [30]. These results are well in agreement with our observation.

Considering, instead, the cases related to events such as major accidents, the percentage of positivity rises sharply. An observational study based on a 3-year period in the United States, showed that out of 706 accidents with 711 fatalities, 42.1% of the drivers had drugs in their biological samples and in 3.8% of the accidents, drugs were a determining factor in the genesis of the accidents [31].

## 5. Conclusions

These final following considerations remark upon the beneficial impact of both the safety training courses and the company policy enforcement; since it is likely that these factors are related to the reduction in the absolute number and percentage of drug users over time.

The creation of an in-house occupational health structure that effectively carries out health surveillance activities appears to be both crucial and necessary for the best and most rapid management of problems related to the workforce and third-party health and safety. A second important aspect is that the reported data are obtained by implementing scrupulous audit procedures, such as those concerning the unpredictability of medical screenings. In addition, the short notice for the convocation shows that the airline in all its aspects (ground and flight) has a very limited number of abusers and that this number decreases over time and with work seniority.

A third aspect of interest, concerning the toxicological field more specifically, is the detection of cross-reactivity in urinary immunochemical screening tests between the drugs of abuse antibodies and some pharmaceutical products, such as anti-inflammatories or antibiotics, as well as foods, or other commonly used substances [23,24].

This aspect has also emerged thanks to the correct implementation of the workforce regulation of workers who threaten third-party safety. According to the policy directives, first level screening must consist of a rapid immunochemical test followed by a confirmatory procedure using GC/MS or LC/MS.

Finally, the examination of the rules and procedures carried out has revealed a further complexity that critically concerns both the commercial and civil aviation industries regarding the OD and the structure through which the issuance of health and fitness statements occurs. In fact, the legislation refers to both the OD and the civil aviation authority (Italian ENAC).

The consistent practice—albeit complex—results from the application of both ENAC Regulation in force and the Legislative Decree no. 81/08 (Consolidated Law on Health and Safety at Work) for which structures concur in the issuing of their statements. This has resulted in an implementation of the health and fitness statement process, as well as an implementation of health surveillance carried out by the OD (as to the aspects of fitness for the specific work) and the AeroMedical Examiner—AME—for the aspects of a Fit-to-Fly certification.

One of the main limitations that prevents a proper comparison of drug prevalence data among air transport personnel from different countries is that there are discrepancies in the national regulations, procedures, typologies of tests, and methods of control regarding the testing of workers in the transport sector. In certain cases, the controls are carried out by Company Health Service Doctors; in others, by public officials or, in the case of road transport in various countries, also by the police. This last fact could explain some of the differences in the percentage seen between professional drivers and pilots in some countries.

## Figures and Tables

**Table 1:** Duties to be subjected to medical examination to exclude drug or psychotropic substance abuse as provided for in Annex 1 to the State-Regions Conference Provision of 2007.

**Use of Poison Gas**	(i.e., maintenance components, base and engine maintenance, manufacturing engineering);
**Airport Ground Operation Vehicles**	(i.e., logistics, look after passengers with special needs, passenger assistance, ramp operations handling, cargo loading, transfers, and airlines);
**Civil Aviation Administration (In Italian Ente Nazionale Aviazione Civile-ENAC) Certification**	(i.e., pilots, flight attendants, aircraft maintenance technicians, aircraft base and engine maintenance, quality systems).

**Table 2:** Threshold concentration (cut-off) in the initial tests and in the confirmatory tests for positivity rates of drug classes in urine (based on the State-Regions Agreement No. 178 of 18 September 2008).

Drug Class	Cut-Off Concentration Initial Tests	Cut-Off Concentration Confirmatory Tests
**Opiates Metabolites** **(morphine, codeine, 6-acetylmorphine)**	300 ng/mL	100 ng/mL
**Cocaine** **and Metabolites**	300 ng/mL	100 ng/mL
**Cannabinoids**	50 ng/mL	15 ng/mL
**Amphetamine-Methamphetamine**	500 ng/mL	250 ng/mL
**MDMA** **-3,4 methylenedioxymethamphetamine (MDA)-Methyldiethanolamine (MDEA)**	500 ng/mL	250 ng/mL
**Methadone**	300 ng/mL	100 ng/mL

**Table 3:** Percentage of positive tests in the analyzed categories of worker populations per year.

	2010	2011	2012	2013	2014	2015	2016	2017	2018	2019
**Workers (ground crew)** total **drug** tests	1804	2290	2888	2768	2711	2841	3365	2882	2839	2923
**Positive sample**	23	36	45	32	27	24	18	16	7	7
**%** Positive **sample**	1.3	1.6	1.6	1.2	1.0	0.8	0.5	0.6	0.2	0.2
**Workers (pilots)** total **drug** tests	1300	1400	1450	1653	1625	1558	1718	1548	1506	1520
**Positive sample**	3	0	1	0	0	0	0	1	0	1
**%** Positive **sample**	0.2	0.0	0.1	0.0	0.0	0.0	0.0	0.1	0.0	0.1
**Workers** (cabin **crew**) total **drug** tests	n.a.	n.a.	n.a.	n.a.	n.a.	n.a.	3143	3913	3878	3549
**Positive sample**	n.a.	n.a.	n.a.	n.a.	n.a.	n.a.	6	8	5	4
**%** Positive **sample**	n.a.	n.a.	n.a.	n.a.	n.a.	n.a.	0.2	0.2	0.1	0.1
Pre-employment total **drug** tests	38	118	203	285	397	945	730	130	798	827
**Positive sample**	1	17	14	28	4	20	18	5	37	16
**%** Positive **sample**	2.6	14.4	6.9	9.8	1.0	2.1	2.5	3.8	4.6	1.9

**Table 4:** Rapid test and confirmatory test positive samples. As shown in the table, out of 614 positive screening tests, only 424 individuals (0.7%) had a positive result at the confirmatory test.

	2010	2011	2012	2013	2014	2015	2016	2017	2018	2019	Total
**Suspected positives detected by rapid test**	49	67	73	74	56	57	75	53	71	38	614
**Positive detected by confirmatory test**	27	53	60	60	31	44	42	30	49	28	424
**% Confirmed tests**	55	79	82	81	55	77	56	56	69	72	69

**Table 5:** Positivity detected in the level 1 tests (screening and confirmatory analysis) divided by analyzed drug.

Analyzed Substances	Rapid Drug Test Positivity Rate with Card Immunochromatography	Confirmatory Drug Test Positivity Rate with GC/MS or LC/MS	% Confirmed Positivity Rate
** Cannabinoids (Delta 9-Tetrahydrocannabinol ** -**THC)**	314	254	80.89
** Cocaine metabolites **	164	153	93.29
** Amphetamine **	40	2	5
** Methamphetamine **	28	3	10.71
** Methadone **	9	7	77.77
** Opioid metabolites **	48	13	27
** MDMA (Ecstasy) **	13	2	15.31
**Total**	616	434	70.45

## Data Availability

Data available on request due to privacy restrictions. The data presented in this study are available on request from the corresponding author.

## References

[1] Peacock A., Leung J., Larney S., Colledge S., Hickman M., Rehm J., Giovino G.A., West R., Hall W., Griffiths P. (2018). Global statistics on alcohol, tobacco and illicit drug use: 2017 status report. Addiction.

[2] Phan H.M., Yoshizuka K., Murry D.J., Perry P.J. (2012). Drug testing in the workplace. Pharmacotherapy.

[3] Pierce A. (2012). Regulatory aspects of workplace drug testing in Europe. Drug Test. Anal..

[4] Presidential Decree No. 309 dated 9 October 1990. https://www.gazzettaufficiale.it/eli/id/1990/10/31/090G0363/sg.

[5] Legislative Decree No. 81 Dated 9 April 2008. https://www.gazzettaufficiale.it/eli/id/2008/04/30/008G0104/sg.

[6] Legislative Decree No. 106 Dated 3 August 2009. https://www.gazzettaufficiale.it/atto/serie_generale/caricaDettaglioAtto/originario?atto.dataPubblicazioneGazzetta=2009-08-05&atto.codiceRedazionale=009G0119&elenco30giorni=false.

[7] State-Regions Conference Provision No. 99/CU 30.10.2007. https://www.gazzettaufficiale.it/eli/id/2007/11/15/07A09622/sg.

[8] State-Regions Agreement REP. ACTS No. 178 Dated 18.09.2008. https://www.gazzettaufficiale.it/atto/serie_generale/caricaDettaglioAtto/originario?atto.dataPubblicazioneGazzetta=2008-10-08&atto.codiceRedazionale=08A07139&elenco30giorni=false.

[9] Lazio Region Decision No. 332 Dated 29th April 2009. https://www.alco-service.it/Doc-MDL/Regione-Lazio.pdf.

[10] Basilicata P., Pieri M., Settembre V., Galdiero A., Della Casa E., Acampora A., Miraglia N. (2011). Screening of several drugs of abuse in Italian workplace drug testing: Performance comparisons of on-site screening tests and a fluorescence polarization immunoassay-based device. Anal. Chem..

[11] National Transportation Safety Board (2014). Drug Use Trends in Aviation: Assessing the Risk of Pilot Impairment Safety Study NTSB/SS-14/01 PB2014-108827.

[12] Li G., Baker S.P., Zhao Q., Brady J.E., Lang B.H., Rebok G.W., DiMaggio C. (2011). Drug violations and aviation accidents: Findings from the US mandatory drug testing programs. Addiction.

[13] Canfield D.V., Dubowski K.M., Chaturvedi A.K., Whinnery J.E. (2011). Drugs and Alcohol in Civil Aviation Accident Pilot Fatalities From 2004–2008.

[14] Civil Aviation Department (2016). Screening Programme for Air Crew in Hong Kong on Psychoactive Substances.

[15] ICAO (2019). ICAO Safety Report 2019.

[16] Guidelines for Workplace Drug Testing in Urine—ISS. https://www.iss.it/doping/-/asset_publisher/Pslu1rErQn8D/content/id/3405463.

[17] Jug M. (2001). Guidelines: Applicability to screening and confirmatory drug tests in the determination of drugs of abuse. Riv. Med. Lab. JLM.

[18] Riboldi L., Porru S., Ferrario M., Feltrin G., Latocca R., Bronzini M. (2009). La prevenzione ed il controllo dell’assunzione di sostanze psicotrope o stupefacenti nei luoghi di lavoro: Una nuova ed importante opportunità per il medico del lavoro. Med. Lav..

[19] ENAC (2020). ENAC Circular of 10.01.20—Implementation of the Regulation: Healthcare Organization and Medical Fitness Certificates for Aeronautical Licenses and Certificates.

[20] EMCDDA (2017). EMCDDA 2017 European Drug Report.

[21] Prime Minister’s Office—Department of Anti-Drug Policies Annual Report to Parliament (Years 2015–2016–2017–2018–2019).

[22] Rosso G.L., Montomoli C., Morini L., Candura S.M. (2017). Seven years of workplace drug testing in Italy: A systematic review and meta-analysis. Drug Test. Anal..

[23] Saitman A., Park H.D., Fitzgerald R.L. (2014). False-positive interferences of common urine drug screen immunoassays: A review. J. Anal. Toxicol..

[24] Smith M.P., Bluth M.H. (2016). Common Interferences in Drug Testing. Clin. Lab. Med..

[25] Qiao S., Xu H., Zhang W., Yang W., Guo D., Wang W., Xu W., Liu Y., Liu G., Cui Y. (2020). Identification of characteristic heroin metabolites in urine based on data-mining technology and multivariate statistics analysis combined with a targeted verification approach for distinguishing heroin abusers. J. Chromatogr. B Anal. Technol. Biomed. Life Sci..

[26] Labat L., Fontaine B., Delzenne C., Doublet A., Marek M.C., Tellier D., Tonneau M., Lhermitte M., Frimat P. (2008). Prevalence of psychoactive substances in truck drivers in the Nord-Pas-de-Calais region (France). Forensic Sci. Int..

[27] Dalén P., Beck O., Bergman U., Björklöv P., Finer D., Garle M., Sjöqvist F. (2000). Workplace drug testing (WDT) likely to increase in Europe. Report from the First European Symposium on WDT including selected abstracts. Eur. J. Clin. Pharmacol..

[28] Verstraete A.G., Pierce A. (2001). Workplace drug testing in Europe. Forensic Sci. Int..

[29] Kazanga I., Tameni S., Piccinotti A., Floris I., Zanchetti G., Polettini A. (2012). Prevalence of drug abuse among workers: Strengths and pitfalls of the recent Italian Workplace Drug Testing (WDT) legislation. Forensic Sci. Int..

[30] Prime Minister’s Office—Department of Anti-Drug Policies Annual Report to Parliament 2012.

[31] Akparibo I.Y., Stolfi A. (2017). Pilot Certification, Age of Pilot, and Drug Use in Fatal Civil Aviation Accidents. Aerosp. Med. Hum. Perform..

